# Derivative Method to Detect Sleep and Awake States through Heart Rate Variability Analysis Using Machine Learning Algorithms

**DOI:** 10.3390/s24134317

**Published:** 2024-07-03

**Authors:** Fabrice Vaussenat, Abhiroop Bhattacharya, Philippe Boudreau, Diane B. Boivin, Ghyslain Gagnon, Sylvain G. Cloutier

**Affiliations:** 1 Department of Electrical Engineering, École de Technologie Supérieure, Université du Québec, Montréal, QC H3C 1K3, Canada; fabrice.vaussenat@lacime.etsmtl.ca (F.V.); abhiroop.bhattacharya.1@ens.etsmtl.ca (A.B.); ghyslain.gagnon@etsmtl.ca (G.G.); 2 Centre for Study and Treatment of Circadian Rhythms, Douglas Mental Health University Institute, Department of Psychiatry, McGill University, Montréal, QC H4H 1R3, Canada; philippe.boudreau@douglas.mcgill.ca (P.B.); diane.boivin@douglas.mcgill.ca (D.B.B.)

**Keywords:** sleep disorders, sleep–wake detection, central nervous system, heart rate variability, RR interval, polysomnography, deep learning, derivative method, IoT, embedded medical device

## Abstract

Sleep disorders can have harmful consequences in both the short and long term. They can lead to attention deficits, as well as cardiac, neurological and behavioral repercussions. One of the most widely used methods for assessing sleep disorders is polysomnography (PSG). A major challenge associated with this method is all the cables needed to connect the recording devices, making the examination more intrusive and usually requiring a clinical environment. This can have potential consequences on the test results and their accuracy. One simple way to assess the state of the central nervous system (CNS), a well-known indicator of sleep disorder, could be the use of a portable medical device. With this in mind, we implemented a simple model using both the RR interval (RRI) and its second derivative to accurately predict the awake and napping states of a subject using a feature classification model. For training and validation, we used a database providing measurements from nine healthy young adults (six men and three women), in which heart rate variability (HRV) associated with light-on, light-off, sleep onset and sleep offset events. Results show that using a 30 min RRI time series window suffices for this lightweight model to accurately predict whether the patient was awake or napping.

## 1. Introduction

Sleep is a biological activity initiated and controlled by the human brain [1]. Sleep helps preserve an individual’s physical and mental health [2]. The human body heals and rebuilds itself during sleep, eliminating metabolic waste accumulated during wakefulness [3]. Sleep also reorganizes memory and promotes the formation of long-term memory [4]. Sleep–wake and circadian disturbances [5] have been associated with increasing the risk of developing serious health problems, including cardiovascular diseases, cognitive impairments, and memory deterioration [6]. The prevalence of sleep-related disturbances is on the rise [7]. It is estimated that roughly 70 millions (approximately 30%) of American adults suffer from various forms of sleep dysfunctions [8]. There is also an increase in the rates of morbidity and mortality among individuals diagnosed with sleep disorders [9]. The primary categories of such sleep disorders include insomnia, sleep-related breathing disorders, central disorders of hypersomnolence, circadian rhythm disorders, parasomnias and sleep-related movement disorders.

In clinical practices, the measurement of physiological signals is a critical step in obtaining quantitative data for the identification of sleep-related disorders. The benchmark for diagnosing such conditions is through polysomnography analysis. Nonetheless, the process of obtaining polysomnography data is laden with challenges [10]; it involves the use of extensive wiring and electrode placement, which is not only time-intensive but may also cause significant discomfort for the individual undergoing the test [11]. Moreover, polysomnography tests are often conducted within specialized sleep center facilities, where unfamiliar surroundings may disturb a patient’s natural sleep patterns, a phenomenon often referred to as the first-night effect, thus potentially skewing the diagnostic results [12]. An alternative approach to circumvent these issues could be the implementation of portable polysomnography devices allowing accurate testing within the familiar confines of a patient’s home, thereby mitigating the first-night effect [13]. Moreover, the traditional manual analysis of polysomnography data can be a laborious task that is susceptible to human biases between individual operators [14]. The push towards automated detection of sleep disorders is driven by the need to overcome such limitations, integrating systematic and objective analysis within the framework of remote monitoring systems. This proposed approach can simultaneously streamline a human- and environment-independent diagnostic process within the patient’s home.

In order to simplify measurements, various techniques have been developed to monitor sleep and wakefulness states. EEG, eye blink, yawning monitoring, breathing rate and heart rate are simple, inexpensive methods for assessing sleep and wakefulness [15]. This information (heart beat) is crucial for parameters such as heart rate variability (HRV) [16]. HRV determination is based on the detection of the QRS complex, which is a group of waves seen on an electrocardiogram, representing ventricular depolarization, allowing the measurement of the interval between two R peaks (RRI) [17]. The entirety of these data (HRV) can be analyzed both in the time and frequency domains, providing vital insights into changes in the central nervous system (CNS) state [17,18]. The CNS regulates daily rhythms, with sympathetic dominance during the day for arousal and parasympathetic dominance at night for rest [19].

With the integration of the Internet of Things (IoT) for personal health and medicine, emerging solutions can combine convenience and accuracy [20]. Many IoT-based devices utilize an accelerometer sensor [21], which measures chest movements to assess respiration. This approach measures the breathing rate [22] (RC), as well as pulse oxygen saturation to determine heart rate and oxygen [23,24,25].

With the increasing adoption of deep learning and classification techniques [26,27], it is now possible to integrate machine and deep learning models into these portable devices. These models, when properly trained, can match or even surpass the accuracy of traditional diagnostic methods, while reducing costs and improving the patient’s experience [28]. There have been technological advancements in portable devices dedicated to monitoring sleep and wakefulness [29,30]. These devices have benefited from the integration of specialized microcontrollers for artificial intelligence (AI), optimizing measurements while maintaining their accuracy and quality [31]. The accuracy of machine learning depends on the feature engineering used [32]. When detecting sleep and wakefulness, machine learning models generally use the EEG signals to achieve the best accuracy [33,34]. RRI is also used, but often in conjunction with EEG waveform values that determine rapid eye movements and indicators of bruxism movements [22].

Derivatives methods are often used to detect small or rapid variations in biosignals [35]. The concept of using the second derivative of the RRI in sleep and wake detection is based on the premise that sleep and wakefulness are distinct physiological states that manifest differently in cardiac activity [36]. The application of the second derivative in sleep and wake detection aligns with broader trends in biomedical signal processing, where advanced mathematical techniques are increasingly employed to extract meaningful information from physiological signal [37].

This study seeks to better understand the complex interrelationship between heart rate variability and sleep patterns. In the specialized domain of electrocardiogram analysis, the application of first and second derivatives is central to the extraction of distinctive features and the identification of cardiac anomalies [38,39]. The second derivative, which represents the acceleration or deceleration of the RRI, can provide valuable insights into rapid changes in heart rate that are indicative of transitions between sleep and wake states.

In our work, we demonstrated that it was possible to accurately determine wakefulness and sleep states from the RRI and its second derivative using a deep learning model. Including the second derivative lets the model consider changes in the RRI’s acceleration [36]. In this paper, the “sleep” label should be considered as “napping” and corresponds to the time during a nap after sleep onset. It may include wakefulness (see Section 2.2.1 for details). This helps ignore temporary states. Such states could cause errors in identifying wakefulness or sleep. Therefore, it improves detection accuracy.

## 2. Materials and Methods

### 2.1. Dataset Details

The Circadian and Sleep Variation dataset serves as the benchmark for this study. This dataset contains nine healthy volunteers (mean age ± SD: 24.6 ± 4.5 years) that were selected for the study 6 men, 3 women and gave informed consent before joining [40].

### 2.2. Data Exploration

Details of the protocol can be found in Boudreau et al., 2011 [40]. Specifically, the protocol for recruiting participants in the study involved a meticulous process. Healthy participants with a normal body mass index provided informed consent before being selected. These participants were required to maintain a regular 8-h sleep pattern for a minimum of two weeks prior to the laboratory study. This adherence was verified through daily telephone check-ins at bedtime (lights off) and wake times (lights on), supplemented by sleep–wake logs and actigraphic recordings to confirm their healthy sleep patterns. The initial phase of the laboratory study commenced in a time isolation suite with a baseline 8-h sleep episode at each participant’s habitual bedtime under controlled lighting conditions in a laboratory setting. Following this, participants underwent a 72-h ultradian sleep–wake cycle in a time isolation suite, consisting of alternating 1 h wake episode followed by 1 h nap opportunities. This design was used to assess circadian rhythms and the impact of various sleep stages on heart rate variability. Throughout this period, RRI and RC were continuously recorded either using a vest with built-in electrodes or via the EKG channel of a polysomnographic recording system. During nap opportunities, sleep was polsysomnograpically recorded and each 30-s epoch scored according to AASM criteria [41]. The R-peaks were extracted from the electrocardiogram (EKG) signal using a validated automatic detection software (VivoLogic, Vivometrics, Ventura, CA, USA) [42]. The EKG data were carefully analyzed to extract R-R intervals (RRI), which were then visually inspected for accuracy and for ectopic beats and artifacts. If necessary, RRI were manually corrected by linear interpolation. This process was carried out by the Centre of Study and Treatment of Circadian Rhythm at McGill University. This preparatory phase and the subsequent experimental procedures ensured that confounding factors were minimized, enabling reliable assessments within the study’s framework. Throughout the entire ultrarapid sleep–wake cycle procedure, 60-min nap opportunity and wake periods were interspersed with events where the light was switched on or off. Some data are missing from the 72 h recordings due to recording problems or noisy data (around 15% of data).

**Figure 1:**
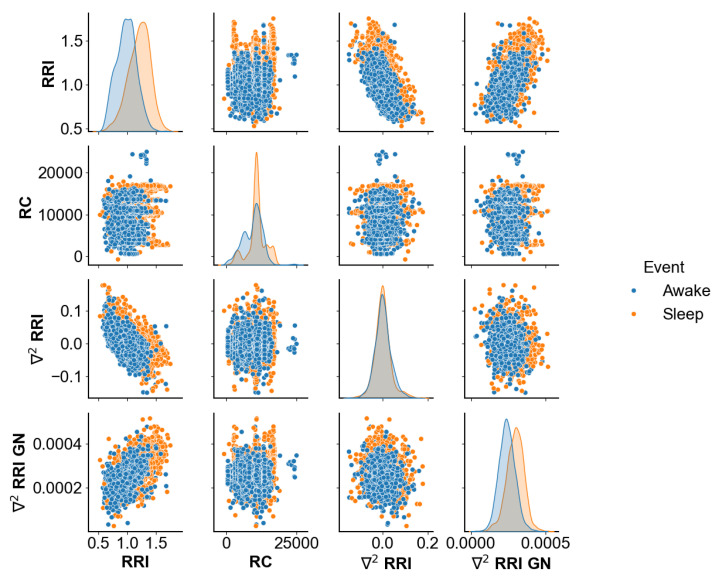
The pair plot distributions highlight the cross-correlations and interrelationships among the key variables such as RR interval, the breathing rate (RC), the classical derivative ($\nabla^{2}RRI$) and the Gregory–Newton ($\nabla^{2}RRI$ GN). It clearly shows that RRI and $\nabla^{2}RRI$ are negatively correlated.

Before applying deep learning to develop a model for classification, we pre-analyzed the RRI for all subjects to determine the occurrence of these events. At this stage, the breathing rate remained a feature included in our model. A correlation map was applied to demonstrate the locality, spread, and skewness groups of the numerical data through their quantiles [43]. The pair plot distributions in Figure 1 highlight the interrelationships among the key variables in the dataset.

#### 2.2.1. Napping and Awake Assessment

Protocol and sleep-related events (i.e., lights on, lights off, and sleep onset) were used to label the RRI and RC signals. All RRI and RC data during wake episodes (i.e., from lights on to the following lights off) were labeled as “awake”. If no sleep occurred during a nap opportunity (i.e., no sleep onset identified), the RRI and RC data were also labeled as “awake”. When sleep occurred during a nap opportunity, RRI and RC signals were labeled as “sleep” from the sleep onset to the following lights on. During this later period (from sleep onset to lights on), the participant could spontaneously wake. This period was always labeled as sleep (see Section 4.1) and its percentage of wakefulness is reported in Table 1. The data presented in Table 1 correspond to available PSG sleep recordings, whereas RRI missing data could be different. We then applied a first- and second-order derivative calculation using the Gregory–Newton method (GNm) and the Pandas library derivative [44,45] on the RRI data. After testing these two methods, we found a high correlation between both, which can be explained by the fact that the derived method used in Python’s Pandas library uses a limited expansion of the Taylor series [45]. The first or second derivative of heart rhythm obtained from an EKG is often employed to detect cardiac anomalies [39,46]. Indeed, the first derivative concerns the rate of change of the HRV parameter or the velocity at which the HRV changes from one moment to another. A high first derivative indicates rapid changes in the intervals between heartbeats, which could reflect a quick response of the autonomic nervous system (ANS) to various stimuli or stress. Conversely, a low first derivative suggests a more stable and less reactive state of the cardiovascular system [47]. The second derivative expresses the acceleration of the RRI parameter. A high absolute value may indicate rapid and potentially unstable fluctuations in cardiac autonomic control, while a low value may indicate greater regularity or stability of these changes [44].

**Figure 2:**
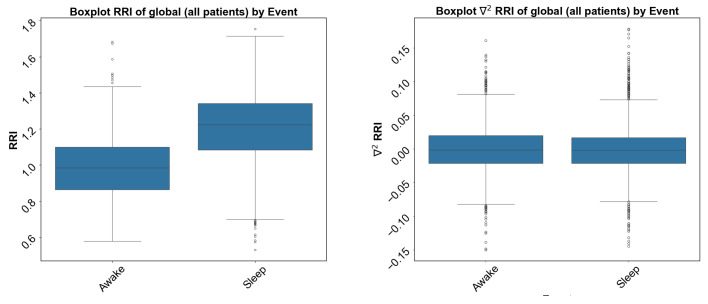
The box plot shows clear differences in the standard deviation and the mean of the RRI and its second derivative ($\nabla^{2}$RRI) for sleep and awake states.

**Figure 3:**
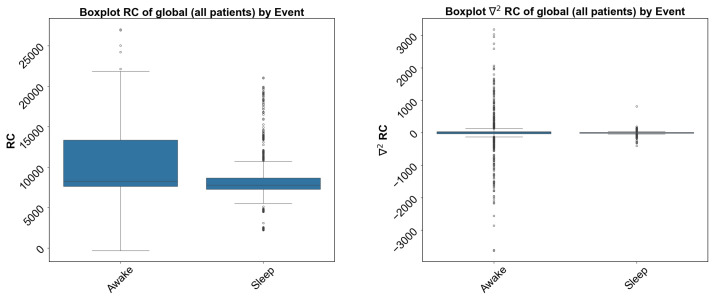
The box plot shows clear differences in the standard deviation and the mean of the respiratory rate from chest and its second derivative ($\nabla^{2}$RC) for sleep and awake states.

Indeed, the first derivative of the RRI, denoted by ∇RRI/∇t can be indicative of a sudden shift in heart rhythm [48]. When the RRI is short, the HRV and the derived equation are positive, meaning the RRI is increasing. On the other hand, when the RRI is long, the HRV and the derivative are negative, explaining the trend of cardiac contraction for the first derivative and the velocity of the trend variable for the second derivative, denoted by $\;\nabla^{2}RRI/\nabla t^{2}$. These parameters allow the quantification of the heart’s response and accurate detection of many abnormal behaviors [39]. When this derivative is applied to the QRS complex, it becomes a more precise indicator of ventricular anomalies because the contractile trend reflects ventricular regularity [49], while the second derivative shows the speed of this trend. Excessive variability in this speed suggests a ventricular rhythmic anomaly with the p-wave, which is the first amplitude of EKG having been absorbed by the derivative calculation [50], thus offering an analysis that presently only pertains to ventricular and contractile analysis. There is a difference in the mean and the standard deviation for the sleep and awake states, as shown in Figure 2 and Figure 3.

#### 2.2.2. Experiment Setup

For the analysis, we considered only two states, sleep and awake. We implemented a neural network with five hidden layers, one input and one output layer for detecting the state of the participant. The input for the neural network was the second derivative of the RRI time series and the RRI time series. Each layer from our deep learning model used a non-linear rectified linear unit activation (Relu). Furthermore, we normalized the input data using min–max normalization [51]. We split the dataset into training, validation and testing according to the ratio 70%, 5% and 25%. To train the neural network, we used a binary cross entropy loss and an Adam optimizer. We kept a constant learning rate of 0.01 throughout the training.

## 3. Results

We ran multiple experiments to measure the classification accuracy of the neural network. All the experiments had the same model architecture and hyperparameters. We used a three-hidden-layer neural network with eight neurons in each layer. We used the Adam optimizer with a learning rate of 0.01. For each experiment, we used different features or combinations of features. We observed that the model had a very low accuracy for predicting the sleep state using only the RRI values. However, if we used RC, the model exhibited an acceptable accuracy of 79% along with an F1 score of 79%. Using the RRI along with the first derivative of HRV resulted in a marginal increase in accuracy. However, an increase in accuracy (around 4%) was achieved when we used the second derivative of the RRI along with the original RRI time series. Table 2 presents a comparative view of the change in model accuracy as we change the input features. To measure the breathing rate, we need to use two sensors, resulting in a complex architecture, while the measurement of RRI uses only one sensor, thereby simplifying the equipment and reducing the cost. Most importantly, previous research on the subject has been able to achieve an accuracy of only 66%, which is much lower than the accuracy achieved by our model [52].

## 4. Discussion

The breathing rate and heart rate variability are critical indicators for assessing the state of the autonomic nervous system (ANS). The ANS, which encompasses the sympathetic and parasympathetic nervous systems, is pivotal in regulating cardiovascular dynamics. Indeed, the HRV reflects the heart’s ability to respond to a wide range of physiological and environmental stimuli [53]. In this context, the use of HRV alone enables sleep and wakefulness to be detected using a simple, low-cost method, without having to take into account the measurement of respiration.

Previous research studies show that HRV varies significantly across different sleep stages and wakefulness, reflecting changes in the autonomic nervous system (ANS) [40,54,55]. Deep sleep, characterized by slower brain waves and reduced muscle activity, is typically associated with increased vagal (parasympathetic) activity [56], resulting in an increased HRV and suggesting a relaxed, restorative state [57]. In contrast, REM (rapid eye movement) sleep, associated with dreaming and increased brain activity, suggests a dominance of sympathetic activity, resulting in a reduced HRV and a state of heightened cardiac activity [58]. During wakefulness, sympathetic activity is more prominent, typically leading to an increased heart rate and reduced HRV [55]. The RRI itself can be a valuable metric for ANS assessment, and its analysis can become more informative when combined with the second derivative of RRI, which helps capture rapid changes in heart rate dynamics and provides a more sensitive and nuanced measure than the heart rate or HRV alone.

By analyzing the patterns of the second derivative in RRIs, it becomes possible to detect more subtle shifts in autonomic nervous system activity accompanying the transition between wakefulness and sleep events. This method could potentially provide a more accurate and responsive means of determining sleep and wake states compared to traditional methods, which usually rely on less dynamic measurement indicators.

Researchers often use the frequency domain analysis of HRV [17]. In this work, we explore the use of the second derivative as a temporal comparison method with the Fourier domain analysis [59]. Incorporating the second derivative of RRI introduces an additional layer of time series analysis. The second derivative, a measure of the rate of change of RRI acceleration, can provide more granular insights into rapid fluctuations of the heart rate. These fluctuations can be indicative of transitions between sleep and wake states, or between different sleep stages. The inclusion of this parameter in a machine learning model for sleep/wake detection could significantly enhance the model’s accuracy. By adding the variability in RRI acceleration in the temporal analysis, the model can become more sensitive to the subtle physiological changes occurring during sleep transitions.

**Figure 4:**
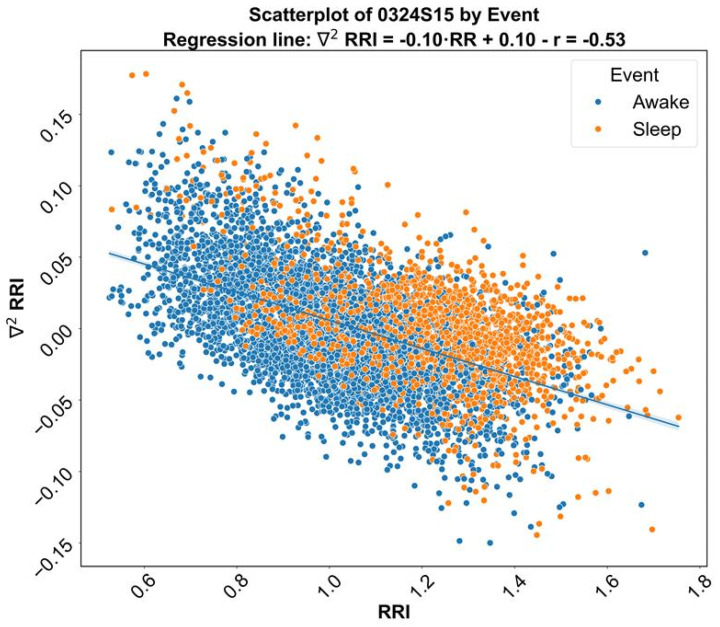
The scatter plot shows that the second derivative and the RRI are negatively correlated.

The scatter plot from Figure 4 shows that the second derivative and the RRI are negatively correlated.

To test the stationary nature of the time series, we ran a Kwiatkowski–Phillips–Schmidt–Shin (KPSS) test. KPSS is a common statistical test for evaluating whether time series data are stationary [60]. The null hypothesis of the test is that the time series is stationary around a mean. Table 3 shows the results of the KPSS analysis for all subjects. We notice a general pattern; the RRI time series has a *p*-value of 0.01, which is lower than the established KPSS threshold of 0.05. As such, we can reject the null hypothesis suggesting that the RRI series is non-stationary. Meanwhile, the second derivative time series has a *p*-value of 0.1, which means that we cannot reject the null hypothesis, suggesting that the RRI series is stationary. The literature shows that statistical models perform better when we use a stationary series, thereby improving the performance of the model [61]. The second derivative of the RRI provides a stationary time series compared to the non-stationary results directly obtained from the RRI, which improves the performance of the model.

These results suggest that the dataset captures measurements from various stages of wakefulness and sleep within the RR interval data. Moreover, we believe this use of the second derivative of the RRI alongside traditional HRV metrics can potentially offer a much more comprehensive understanding of the underlying autonomic modulation, providing a deeper understanding of the complex interplay between the cardiovascular system and sleep physiology. It is a significant advancement in the field of biomedical signal processing, where sophisticated analytical techniques are increasingly being employed to extract meaningful insights from physiological data for clinical and research purposes. Future work should focus on isolating these events. As a future scope of work, employing HRV frequency domain analysis is recommended. This technique will help link the observed results to validated drowsiness indicators. For a comprehensive understanding, readers are referred to articles on HRV time and frequency domain measures, which provide essential background and methodologies for this approach.

### 4.1. Strengths and Limitations

In this paper, we demonstrated that a neural network with a small number of parameters can be used to detect the sleep or wakefulness states using the second derivative of the RRI. Our approach is cost effective, non-invasive and can be deployed in embedded devices using quantization. Moreover, we showed that the use of the second derivative improves the accuracy of the model compared to using the raw RRI signal. During naps, participants presented wake epochs after sleep onset, prior to lights on. These data were also labeled as sleep, since sleep onset had occurred and napping conditions were in effect. This limitation could have affected the accuracy rate. In this study, we employed a conventional machine learning approach to divide the data into training and test sets. Consequently, it is possible for a single participant’s data to be included in both the training and test datasets simultaneously. Thus, the samples in the test set could be from the same sessions as those from the training set.

## 5. Conclusions

In this paper, we proposed a deep learning model to accurately predict the sleep and awake states of a subject using only the RR interval measurements. We established that the second derivative, which represents the acceleration or deceleration of the RR interval velocity, provides valuable insights into rapid changes in heart rate variability that are indicative of transitions between sleep and wake states. As such, including this information as an embedded feature for the neural network yields significant improvements in model performance.We believe that the use of such neural networks with sensors will potentially allow a better monitoring of sleep patterns.

## Figures and Tables

**Table 1:** Duration of wakefulness (in min. and %) following sleep onset.

P.	Mean (min.)	SD (min.)	Mean (%)	SD (%)
S07	3.28	5.57	9.0	15.9
S08	3.03	5.96	8.8	17.9
S09	3.18	7.54	7.5	17.8
S10	2.08	2.57	5.6	6.5
S11	3.30	3.99	8.2	10.5
S12	9.16	12.85	28.5	35.1
S13	9.60	15.09	23.8	23.8
S14	8.65	9.65	21.3	26.3
S15	9.00	14.03	22.0	33.5

**Table 2:** Effect of input features on the classification accuracy.

	ClassificationMetrics(%)			
	Accuracy	Precision	Recall	F1Score
RC	58.31	59	58	58
RRI	79.05	79	79	79
RRI+∇RRI	79.86	80	80	80
$RRI+\nabla^{2}RRI$	83.28	83	83	83

**Table 3:** KPSS test results.

	StatisticalMetrics		
**Subject**	**Data**	Statistic	***p*** **-Value**
RRISeries	S07	1.636	0.01
	S08	2.09	0.01
	S09	2.012	0.01
	S10	2.135	0.01
	S11	1.916	0.01
	S12	0.92	0.01
	S13	2.41	0.01
	S14	1.52	0.01
	S15	1.63	0.01
$\nabla^{2}RRISeries$	S07	0.063	0.1
	S08	0.074	0.1
	S09	0.162	0.1
	S10	0.087	0.1
	S11	0.054	0.1
	S12	0.267	0.1
	S13	0.131	0.1
	S14	0.132	0.1
	S15	0.239	0.1

## Data Availability

The data for the project were provided by the Centre for Study and Treatment of Circadian Rhythms, Douglas Mental Health University Institute, Department of Psychiatry, McGill University. The experimental data used to train the machine learning algorithms cannot be shared publicly because participants did not agree. Therefore, for ethical and confidentiality reasons, the authors cannot provide public access to them. Nevertheless, the authors agree to make data and materials supporting the results or analyses available for the investigation of scientific integrity if necessary. The code will be made available upon reasonable request.
